# Supplementary material for: Evidence supported by Mendelian randomization: impact on inflammatory factors in knee osteoarthritis
Source: Front Med (Lausanne). 2024 May 28;11:1382836. doi: 10.3389/fmed.2024.1382836 (PMC11165061; doi:10.3389/fmed.2024.1382836)
Supplement: Supplementary file 1 [file Data_Sheet_1.ZIP › data/GCST90274825_5e6/825.leaveoneout.pdf]

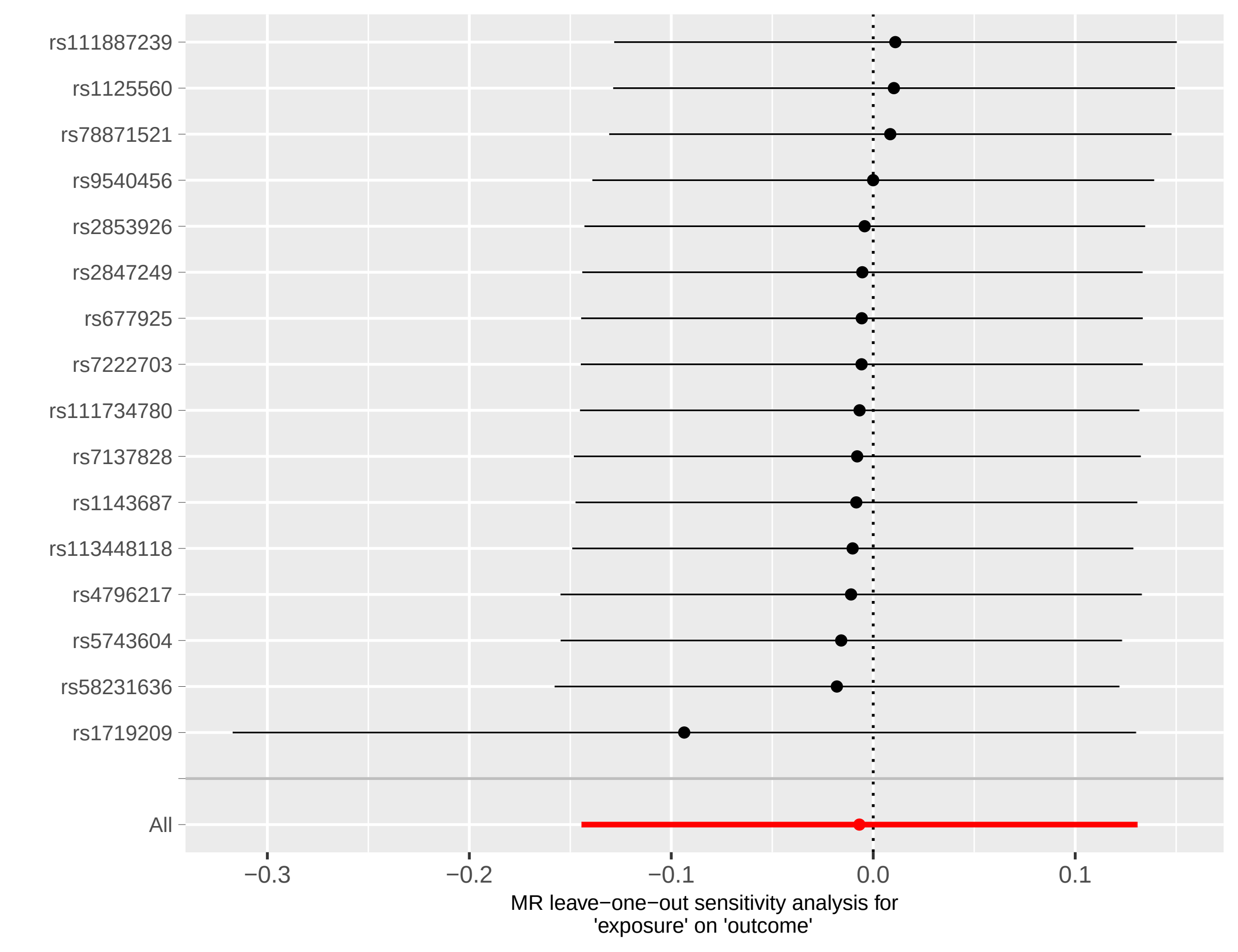

rs111887239
rs1125560
rs78871521
rs9540456
rs2853926
rs2847249
rs677925
rs7222703
rs111734780
rs7137828
rs1143687
rs113448118
rs4796217
rs5743604
rs58231636
rs1719209
All
−0.3
−0.2
−0.1
0.0
0.1
MR leave−one−out sensitivity analysis for
'exposure' on 'outcome'
